# Exploring the Relationship between E-Cadherin and β-Catenin Cell Adhesion Proteins and Periacinar Retraction Clefting in Prostatic Adenocarcinoma

**DOI:** 10.3390/diagnostics14050511

**Published:** 2024-02-28

**Authors:** Rinë Limani, Cvjetko Lež, Božo Krušlin

**Affiliations:** 1 Faculty of Medicine, University of Prishtina “Hasan Prishtina”, 10000 Prishtina, Kosovo; 2 Institute of Anatomical Pathology, University Clinical Center of Kosovo, 10000 Prishtina, Kosovo; 3 Faculty of Dental Medicine and Health Osijek, Josip Juraj Strossmayer University of Osijek, 31000 Osijek, Croatia; 4 Department of Pathology, Zabok General Hospital, 49210 Zabok, Croatia; 5 “Ljudevit Jurak” Department of Pathology and Cytology, Sestre Milosrdnice University Hospital Center, 10000 Zagreb, Croatia; bozo.kruslin@mef.hr; 6 Department of Pathology, School of Medicine, University of Zagreb, 10000 Zagreb, Croatia

**Keywords:** periacinar clefts, E-cadherin, β-catenin, prostate, adenocarcinoma

## Abstract

Background: Periacinar retraction clefts represent a histopathological criterion supporting the diagnosis of prostatic adenocarcinoma. The origin of these clefts in prostatic adenocarcinoma remains unclear. Exploring the established functions of E-cadherin and β-catenin as intercellular adhesion proteins, and aiming to elucidate the origin of periacinar retraction clefting, we conducted a correlation study between the immunohistochemical expression of E-cadherin and β-catenin and the presence of periacinar retraction clefts in prostatic adenocarcinoma. Methods: We examined 53 cases of morphologically diagnosed prostatic adenocarcinoma, assessing both the neoplastic and adjacent nonneoplastic prostatic tissues for the existence and degree of periacinar retraction clefts. Additionally, we analyzed the immunohistochemical expression of E-cadherin and β-catenin proteins in prostatic tissue and explored their correlation with periacinar retraction clefts, and Gleason score, Grade Group, preoperative serum prostate specific-antigen (sPSA) levels, surgical margin status, and Tumor, Node, Metastasis (TNM) stage in prostatic adenocarcinoma. Results: Our study confirms that periacinar retraction clefting is significantly more extensive in prostatic adenocarcinoma than in nonneoplastic prostatic tissue (*p* < 0.001). We report a decreased expression of E-cadherin and β-catenin immunostaining in prostatic adenocarcinoma and a negative correlation with Gleason score and Grade Group. Periacinar retraction clefting positively correlated with E-cadherin and β-catenin ((rho = 0.350; *p* = 0.010) and (rho = 0.340; *p* = 0.012)) immunostaining in prostatic adenocarcinoma. Conclusions: Periacinar retraction clefts stand out as a dependable criterion in the diagnosis of prostatic adenocarcinoma. E-cadherin and β-catenin proteins are potential markers indicative of tumor progression and invasiveness in prostatic adenocarcinoma. Our discovery of a positive correlation between immunostaining of E-cadherin and β-catenin proteins and periacinar retraction clefts in prostatic adenocarcinoma aligns with the notion that periacinar retraction clefting is more characteristic of Gleason Grade3 pattern in prostatic adenocarcinomas, whereas the immunohistochemical expression of E-cadherin and β-catenin shows a decrease with increasing histopathological tumor grade.

## 1. Introduction

The histopathological diagnosis of prostatic adenocarcinoma relies on a combination of various histological features. Three established diagnostic criteria for prostatic adenocarcinoma are the infiltrative growth pattern, the absence of a basal cell layer, and the presence of macro nucleoli [1]. Periacinar retraction cleftings, also known as retraction artifacts, periacinar halos, or cleft-like spaces, play a significant role in supporting the histopathological diagnosis of prostatic adenocarcinoma [1,2,3,4,5]. These clefts manifest as neoplastic cells “pull away” from the surrounding stroma, creating halos around the acini. Krušlin et al. [3], observed that periacinar retraction clefting, accounting for more than 50% of the circumference in at least 50% of suspicious glands, serves as a reliable criterion for diagnosing prostatic adenocarcinoma.

Various hypotheses have been proposed concerning the origin of retraction clefts and the biological mechanisms that lead to clefting in tumor specimens. Irregularities in the basement membrane, involving the altered expression of extracellular matrix proteins and collagenases essential for invasion, as well as stromal changes and the absence of basal cells have been suggested to be related to the origin of periacinar clefts. Moreover, the so called retraction artifacts are suggested to represent an early stage of lymphovascular invasion in breast carcinoma. Ulamec et al. [4], utilizing the D2-40 antibody to highlight lymphatic endothelium to differentiate between authentic lymph vessels or lymphovascular invasion and periacinar retraction clefts, observed a significant reduction in the number of lymph vessels in prostatic adenocarcinoma compared to the adjacent nonneoplastic prostatic tissue. As a result, they concluded that retraction clefts should be regarded as a distinct entity in prostatic adenocarcinoma.

Cadherins exert a fundamental influence on morphogenic processes during development. E-cadherin, encoded by the Cdh1 gene, stands as the pioneering member of the cadherin superfamily. It forms complexes with actin cytoskeleton and cytoplasmic catenin molecules, essential for upholding the functional attributes and structural integrity of epithelial tissues [6,7,8].

E-cadherin stands out as a crucial molecule in cell–cell adhesion within epithelial tissues. It plays a vital role in the establishment and preservation of normal epithelia. The downregulation of E-cadherin expression is recognized as a primary molecular event responsible for impairing cell–cell adhesion [6,7,8].

β-catenin participates in organogenesis and tissue morphogenesis, exerting a pivotal influence on the control of cadherin-mediated cell recognition and adhesion. It serves as the regulator of the cadherin–catenin complex, facilitating signal transduction within intercellular adhesions. In addition, the Wnt/β signaling facilitates the expression of genes involved in extracellular matrix remodeling and turnover [9,10].

As cadherins and catenins are key regulators of cell–cell adhesion and play a crucial role in governing morphological differentiation and cellular proliferation, the disruption of their intercellular function in cancer is reported to enable malignant cells to evade their original location, break down the extracellular matrix, adopt a more mobile phenotype, and initiate the invasion and metastasis. E-cadherin and β-catenin immunohistochemical expression is strong and diffuse in normal prostatic epithelial tissue [6,7,8,9,10].

To elucidate the origin of periacinar retraction clefting, and taking into account the established roles of E-cadherin and β-catenin as intercellular adhesion proteins and their involvement in extracellular matrix remodeling and turnover, we analyzed and correlated the expression of E-cadherin and β-catenin with the presence and extent of periacinar retraction clefting in prostatic tissue, as well as with Gleason score (GSC), International Society of Urological Pathology (ISUP) Grade Group, preoperative prostate-specific antigen (sPSA), surgical margin status, and Tumor, Node, Metastais (TNM) staging in prostatic adenocarcinoma.

## 2. Patients and Methods

### 2.1. Patients

The research was conducted using archival tissue specimens of prostatic adenocarcinoma and the adjacent nonneoplastic prostatic tissue, obtained through radical prostatectomy at the Department of Pathology “Ljudevit Jurak” of the Clinical Hospital Center “Sestre milosrdnice” in Zagreb, Croatia.

To safeguard patient confidentiality, patient identifiers were substituted with study numbers. A total of 53 prostate samples, morphologically diagnosed as prostatic adenocarcinoma, underwent analysis. In recognition of periacinar retraction clefting being a more prevalent morphological feature in Gleason Grade 3 pattern of prostatic adenocarcinoma [3,4,5,11], our sample selection intentionally included a higher percentage of GSC 6 (3+3) samples, rather than being randomly chosen. Prostates with low-volume cancer were included in the study. Inclusion criteria for a tissue block comprised the presence of identifiable prostatic adenocarcinoma and the availability of adjacent nonneoplastic prostatic tissue. None of the patients had undergone preoperative hormonal therapy or radiotherapy. This retrospective study received approval from the Ethical Committee of the School of Medicine, University of Zagreb, Croatia.

### 2.2. Methods

Sections were cut to 5µm thickness from paraffin blocks containing prostatic tissue fixed in 10% buffered formaldehyde. Slides were subsequently deparaffinized and stained with hematoxylin and eosin (H&E). The presence and extent of periacinar retraction clefting were determined using light microscopy (Nikon, Tokyo, Japan) under high-power field magnification (400×), and a minimum of 30 neoplastic and 30 nonneoplastic glands were assessed. Periacinar retraction clefting was graded as a percentage of gland circumference separated from the stroma in three categories, as previously described [3]:

- Group 1: Glands without clefts or with clefts affecting less than 50% of the circumference;
- Group 2: Glands with clefts that affect more than 50% of the circumference in less than 50% of examined glands;
- Group 3: Glands with clefts that affect more than 50% of the circumference in 50% or more of the examined glands.

Tumor grade and the TNM were determined according to the latest guidelines [1].

#### 2.2.1. Immunohistochemistry

Immunohistochemical analysis of the expression of E-cadherin (code M3612, clone NCH-38, dilution 1:50) and β-catenin (code M3539, clone β-catenin-1, dilution 1:200) cell adhesion proteins in prostatic adenocarcinoma and in the adjacent nonneoplastic prostatic tissue was performed using an EnVision Flex system (Dako, Glostrup, Denmark) on a Dako TechMate^TM^ immunohistochemical autostainer (Dako, Glostrup, Denmark). Primary antibodies were purchased from Dako, Glostrup, Denmark. Breast cancer tissue was used as a positive control and Mouse IgG1 (code X0931) as a negative control.

#### 2.2.2. Scoring

Immunohistochemical staining results for both markers were assessed by considering the intensity of cell staining and the approximate percentage of positive cells using light microscopy under high-power field magnification (400×). The intensity of staining was graded semi-quantitatively as 0 (no staining), 1+ (weak), 2+ (moderate), and 3+ (strong). The cutoff for the percentage of positive cells was set to 70% for both markers. The immunostaining for both markers was considered negative (intensity score 0 and 1 and <70% of positive cells), weak (intensity score 1 and >70% of positive cells, and intensity score 2 and 3 and <70% of positive cells), or positive (intensity score 2 and 3 and >70% of positive cells).

### 2.3. Statistical Methods

Data are presented in tables. Quantitative values are shown through medians and corresponding interquartile ranges. Differences in periacinar retraction clefting in relation to other clinical parameters regarding categorical variables were analyzed with the chi-square test, and differences in quantitative variables were analyzed with the Kruskal–Wallis test.

Spearman’s and Kendall’s tau_b (for nominal variables) correlation coefficients were calculated to assess the correlation of E-cadherin and β-catenin immunohistochemical expression with periacinar retraction clefting, GSC, Grade Group, preoperative sPSA, surgical margin status, and the TNM staging. All *p*-values below 0.05 were considered significant.

Statistical software IBM SPSS Statistics version 21 was used in all statistical procedures. All samples were independently reviewed by two observers.

## 3. Results

Descriptive statistics of the patients are summarized in Table 1. More than half of the patients (56.6%) had a GSC of 6 (3+3), and nearly two-thirds of patients (66%) had T2N0Mx stage. Grade Group 1 was the most frequent group, making up 56.6% of our sample (Table 1).

Forty tumors (75.5%) were confined to the prostate and thirteen (24.5%) patients had positive surgical margins, with the tumor spreading through the prostatic capsule. The median (interquartile range—IQR) age of patients was 64.0 (61.0–67.0) years and the median preoperative sPSA value was 9.0 (6.5–12.8) ng/mL.

**Figure 1:**
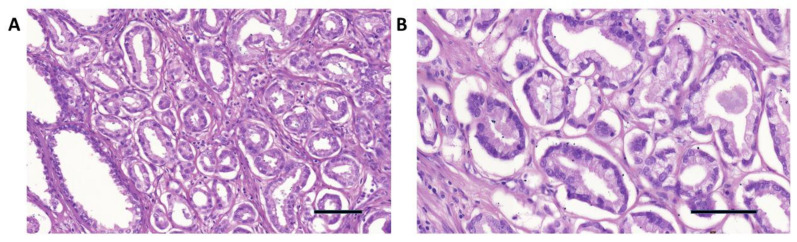
(**A**) Extensive periacinar retraction clefting in Grade Group 1 and GSC 6 (3+3) prostatic adenocarcinoma (center and upper right) and no clefting in the adjacent nonneoplastic glands (lower left) H&E (100×). (**B**) Extensive periacinar retraction clefting in Grade Group 1 and GSC 6 (3+3) prostatic adenocarcinoma H&E (200×). Scale bar: 100 µm.

Periacinar retraction clefting was more of a characteristic feature of GSC 6 (3+3) prostatic adenocarcinoma. Namely, 28 (86.66%) out of 30 tumors with GSC 6 (3+3) in our study sample had clefts that affect more than 50% of the circumference in 50% or more of the examined glands (Table 2). Nevertheless, the most frequent group with periacinar retraction clefting in prostatic adenocarcinoma was Group 3, i.e., glands with clefts that affect more than 50% of the circumference in 50% or more of the examined glands (52.83%) (Figure 1A,B and Table 3), suggesting a noteworthy prevalence of extensive clefting in the studied tumor samples. Group 1, i.e., glands without clefts or with clefts affecting less than 50% of the circumference, was present in 32 (60.37%) samples in the adjacent nonneoplastic prostatic tissue (Table 3). Periacinar retraction clefting was more extensive in prostatic adenocarcinoma samples than in the adjacent nonneoplastic prostatic tissue (*p* < 0.001).

**Figure 2:**
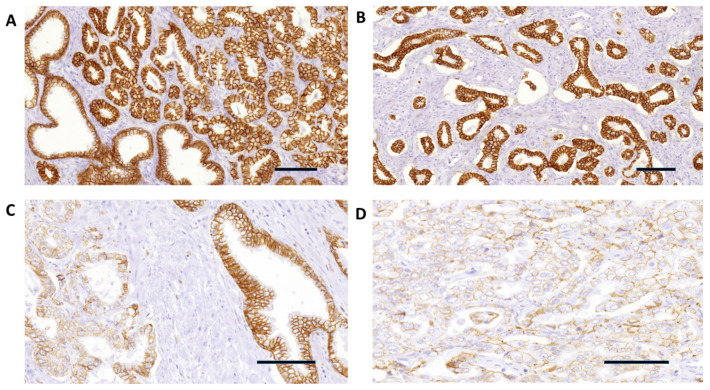
(**A**) E-cadherin strong immunohistochemical expression in Grade Group 1 and GSC 6 (3+3) prostatic adenocarcinoma (upper right), and in the adjacent nonneoplastic prostatic glands (lower left) (100×). (**B**) E-cadherin strong and diffuse immunohistochemical expression in Grade Group 1 and GSC 6 (3+3) prostatic adenocarcinoma glands with extensive periacinar retraction clefting (40×). (**C**) E-cadherin negative expression in Grade Group 3 and GSC 7 (4+3) prostatic adenocarcinoma (left) and strong expression in the adjacent nonneoplastic glands (right) (200×). (**D**) E-cadherin negative immunohistochemical expression in Grade Group 4 and GSC 8 (4+4) prostatic adenocarcinoma (400×). Scale bar: 100 µm.

**Figure 3:**
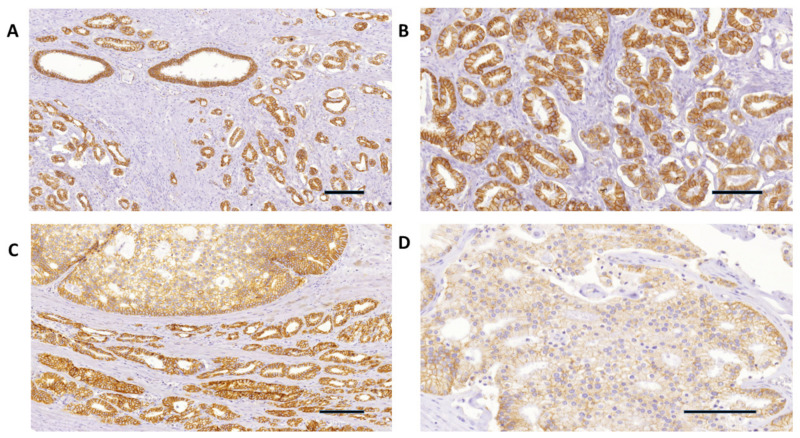
(**A**) β-catenin strong immunohistochemical expression in Grade Group 1 and GSC 6 (3+3) prostatic adenocarcinoma (lower left and right), and in the adjacent nonneoplastic prostatic glands (upper left) (20×). (**B**) β-catenin strong and diffuse immunohistochemical expression in Grade Group 1 and GSC 6 (3+3) prostatic adenocarcinoma glands with extensive periacinar retraction clefting (100×). (**C**) β-catenin weak expression in Gleason Grade 4 prostatic adenocarcinoma (upper side) and strong expression in the adjacent Gleason Grade 3 glands (40×). (**D**) β-catenin negative immunohistochemical expression in Grade Group 4 and GSC 8 (4+4) prostatic adenocarcinoma (400×). Scale bar: 100 µm.

The expression pattern for E-cadherin and β-catenin, as determined using immunohistochemistry, was predominantly membranous and weakly cytoplasmic for E-cadherin and predominantly membranous and weakly to moderate cytoplasmic for β-catenin, in both prostatic adenocarcinoma and the adjacent nonneoplastic prostatic tissue. Figure 2A–D and Figure 3A–D and Table 3 show the pattern of E-cadherin and β-catenin immunostaining in prostatic adenocarcinoma and in the adjacent nonneoplastic prostatic tissue. Strong positive immunostaining of E-cadherin and β-catenin (intensity score 2 and 3 and >70% of positive cells) was observed more frequently in the samples of the adjacent nonneoplastic tissue than in prostatic adenocarcinoma (*p* < 0.00001). E-cadherin staining was weak and negative in 92.46% of prostatic adenocarcinoma samples as opposed to 9.43% of the adjacent nonneoplastic prostatic tissue (Table 3). β-catenin immunostaining in prostatic adenocarcinoma was weak and negative in 47.26% of samples as opposed to 7.55% of samples in the adjacent nonneoplastic prostatic tissue (Table 3).

Table 4 summarizes the correlation of E-cadherin and β-catenin expression with GSC, Grade Group, preoperative sPSA, surgical margin status, and the TNM staging. Negative correlations were found between the E-cadherin and β-catenin immunostaining in prostatic adenocarcinoma with GSC ((rho = −0.323; *p* = 0.025) and (rho = −0.750; *p* = 0.031)) and Grade Group ((rho = −0.63; *p* = 0.038) and (rho = −0.56; *p* = 0.019)). No statistically significant correlations were found between E-cadherin and β-catenin immunostaining and the surgical margin status ((rho = −0.550; *p* = 0.345) and (rho = −0.390; *p* = 0.293)), preoperative sPSA ((rho = 0.06; *p* = 0.999) and (rho = 0.11; *p* = 0.998)); T stage ((rho = −0.720; *p* = 0.111) and (rho = −0.460; *p* = 0.143)), and N status ((rho = −0.970; *p* = 0.696) and (rho = −0.680; *p* = 0.545)).

Periacinar retraction clefting positively correlated with E-cadherin and β-catenin immunostaining in prostatic adenocarcinoma ((rho = 0.350; *p* = 0.010) and (rho = 0.340; *p* = 0.012)) (Table 4). In the adjacent nonneoplastic prostatic tissue, periacinar retraction clefting was not extensive; correlations with E-cadherin and β-catenin, as presented in Table 5, were (rho = −0.310; *p* = 0.542) and (rho =−0.006; *p* = 0.664), respectively.

## 4. Discussion

Retraction clefts have been identified as a notable feature in several human carcinomas, including prostatic adenocarcinoma. In prostatic adenocarcinoma, the phenomenon of retraction clefting refers to the formation of cleft-like spaces around acini within the tumor mass.

Young et al. [11], discussing the association between Gleason Grade 3 pattern in prostatic adenocarcinoma and prominent periacinar retraction clefts, proposed that this occurrence was likely an artifact. In line with earlier research outcomes [3,4,5], our investigation establishes that periacinar retraction clefting is evident in neoplastic glands within prostatic adenocarcinoma. This observation is particularly notable when the clefts impact over 50% of the circumference in 50% or more of the examined glands (Figure 1A,B, Table 2 and Table 3). Favaro et al. [5], in their study of periacinar retraction clefts in prostatic adenocarcinoma, reported that periacinar retraction clefting was more extensive in GSC 6 (3+3), with clefting affecting up to 50% of gland circumference in four samples (28.0%) and clefting affecting more than 50% of gland circumference in ten samples (72.0%) [6]. In our study group, we also report GSC 6 (3+3) to have the most extensive periacinar retraction clefting, with clefts affecting more than 50% of the gland circumference in more than 50% of the examined glands presenting in 86.7% of GSC 6 (3+3) prostatic adenocarcinomas (Table 2).

Abnormalities in the basement membrane, loss of the adhesion factors, and altered expression of extracellular matrix proteins or enzymes such as collagenases, along with stromal changes and the absence of basal cells are proposed to be associated with the occurrence of retraction clefting in prostatic adenocarcinoma [4,5,6]. Retraction cleftings are also considered an early stage of lymphocapillary invasion, where the transformation of mesenchymal cells into endothelial cells has not yet been completed; therefore, it is considered that as a result, they represent genuine spaces or a “pseudoretraction artifact” surrounding the tumor cells [12,13,14].

Nevertheless, despite the well-established correlation between retraction clefting and malignant epithelial glands, its origin remains unknown.

In our study, we analyzed the immunoexpression of E-cadherin and β-catenin cell adhesion proteins in periacinar retraction clefting in prostatic adenocarcinoma and in the adjacent nonneoplastic prostatic tissue and we correlated it with GSC, Grade Group, preoperative sPSA, surgical margin status, and the TNM staging in prostatic adenocarcinoma. We considered that the roles of E-cadherin and β-catenin in cell-cell adhesion and extracellular remodeling could be crucial in understanding this process.

E-cadherin, a transmembrane glycoprotein, is a key player in maintaining cell–cell adhesion and tissue integrity. In normal prostate tissue, E-cadherin expression is typically high, contributing to the cohesive nature of epithelial cells. However, during the progression of prostate cancer, E-cadherin expression can become dysregulated. Reduced levels of E-cadherin are observed in prostatic adenocarcinoma, correlating with increased tumor aggressiveness and metastatic potential [15,16,17]. The loss or downregulation of E-cadherin expression in prostate cancer cells diminishes cell–cell adhesion, leading to decreased cohesion between tumor cells. This reduction in cell–cell adhesion allows cancer cells to dissociate from the primary tumor mass [7]. Consequently, the detached cancer cells acquire the ability to invade the surrounding extracellular matrix, which we considered to potentially play a role in periacinar cleft formation in the tumor microenvironment of prostatic adenocarcinoma.

Moreover, dysregulation of β-catenin signaling can affect the expression of genes involved in extracellular remodeling [9,10]. In the context of retraction clefting, we considered that aberrant β-catenin signaling may promote extracellular matrix degradation and the creation of clefts in the surrounding stroma of prostatic acini in prostatic adenocarcinoma.

The interplay between catenins and cadherins is pivotal for the proper functioning of cell–cell adhesion complexes within epithelial tissue. E-cadherin, facilitated by catenins, establishes a connection with the actin network at the cell–cell adhesion junction, and β-catenin plays a crucial role as one of the main participants in this E-cadherin-mediated cell–cell communication [6,7,8]. β-catenin is also a key signaling molecule in the Wnt and phosphatidylinositol 3-kinase/Akt cancer signaling pathways [9,10].

E-cadherin and β-catenin proteins are described as valuable tumor markers, as their altered expression has been shown to correlate with increased tumor aggressiveness and dedifferentiation in human cancers, including prostatic adenocarcinoma. A decrease in E-cadherin expression is described to correlate with advanced GSC and advanced pathologic stage in prostatic adenocarcinoma [7,15,16,17,18,19]. Lower E-cadherin expression is reported to be related to worse overall-survival and disease-free survival (HR 3.69, 95%CI 1.18–11.50; HR 5.90, 95%CI 1.40–24.81) in the pT3b group of prostatic adenocarcinoma in the study of Ferreira et al. [15]. Jaggi et al. [16], reported downregulation of E-cadherin in GSC 7–10 prostatic adenocarcinoma compared with GSC ≤ 6 (*p* = 0.015), suggesting a significant association between decreased E-cadherin and increasing grade. They also found an association between decreasing membranous β-catenin expression in prostatic adenocarcinoma and increasing GSC (*p* = 0.025). Moreover, they report β-catenin nuclear immunolocalization in poorly differentiated cancer cells and a correlation between higher GSC of 7–10 and nuclear β-catenin expression in prostatic adenocarcinoma (*p* = 0.0001) [16]. Several other studies have reported a β-catenin shift from cell membrane to cytoplasm and nucleus with increasing grade in prostatic adenocarcinoma [20,21].

E-cadherin immunostaining was predominantly membranous and weakly cytoplasmic, whereas β-catenin immunostaining was predominantly membranous and weakly to moderate cytoplasmic, in both prostatic adenocarcinoma and the adjacent nonneoplastic prostatic tissue in our study. We did not notice any difference in the immunolocalization of the staining (membranous vs. cytoplasmic) between prostatic adenocarcinoma and the adjacent nonneoplastic prostatic tissue; therefore, we did not consider this pattern of staining as aberrant. Statistical analyses of our results confirm previously reported studies on the loss of E-cadherin expression in prostatic adenocarcinoma and the negative correlation between E-cadherin immunostaining and GSC (Table 3) [11,15,16,17,18,19]. Additionally, we also report a negative correlation between E-cadherin staining and Grade Group in prostatic adenocarcinoma.

β-catenin immunostaining was well preserved in 52.83% of prostatic adenocarcinomas in our samples, and there was no statistically significant difference in the β-catenin immunostaining between prostatic adenocarcinoma and the adjacent prostatic nonneoplastic tissue (Table 3). Our data are in accordance with previously reported studies on the decreased expression of β-catenin immunostaining in a certain percentage of prostatic adenocarcinomas [11,20,21]. Furthermore, we report a negative correlation between β-catenin immunostaining and GSC and Grade Group in prostatic adenocarcinoma (Table 4). Yet, there are inconsistent and conflicting data in the literature regarding β-catenin immunolocalization and expression in the prostatic tissue. Several studies have reported β-catenin nuclear immunolocalization in normal and benign hyperplastic tissue of the prostate, and a decrease in membranous and nuclear β-catenin with increasing GSC in prostatic adenocarcinoma [20,21].

We did not detect any nuclear β-catenin staining in prostatic tissue, and we report β-catenin membranous and weak-to-moderate cytoplasmic immunostaining in both prostatic adenocarcinoma and the adjacent nonneoplastic prostatic tissue in our samples. Similar findings are reported by several other authors [22,23]. Bismar et al. [22], in their study comparing β-catenin immunoexpression between colorectal and prostatic adenocarcinoma, could not demonstrate nuclear β-catenin staining in prostatic adenocarcinoma. Conflicting reports in the literature on β-catenin expression in prostatic tissue can be a result of various methodologies, clones, and immunohistochemical protocols used for its detection. Our results suggest a potential role of E-cadherin and β-catenin proteins in tumor progression in prostatic adenocarcinoma. 

Positive correlation between periacinar retraction clefting and the immunohistochemical expression of E-cadherin and β-catenin in our study corresponds to periacinar retraction clefting being more of a characteristic feature for Gleason Grade 3 pattern in prostatic adenocarcinomas. On the other hand, E-cadherin and β-catenin immunohistochemical expression decreased with increasing histopathological tumor grade and therefore their expression was mainly preserved in the glands of low-grade tumors of GSC 6 (3+3), which had the most extensive periacinar halos and represented more than 50% of our sample.

When considering the limitations of our study, we acknowledge the small sample size. Moreover, the intentional inclusion of a higher percentage of GSC 6 (3+3) samples in the study design introduces a potential source of bias as it overemphasizes low-grade tumors. We consider that a larger and more diverse sample size would enhance the generalizability of the findings.

## 5. Conclusions

Periacinar retraction clefting can be used as a reliable criterion in making the diagnosis of prostatic adenocarcinoma. E-cadherin and β-catenin proteins may play a role as potential markers for tumor progression and invasiveness in prostatic adenocarcinoma. Our findings suggest that periacinar retraction clefts are not directly related to the cell–cell adhesion phenomena and the extracellular matrix changes mediated by E-cadherin and β-catenin proteins in prostatic adenocarcinoma, and that a relation between other potential stromal changes and periacinar retraction clefting should be further investigated.

## Figures and Tables

**Table 1:** Descriptive statistics of patient characteristics: categorical variables: Gleason score (GSC), Grade Group, surgical margin status, Tumor, Node, Metastasis (TNM) staging, Tumor (T) stage, Node (N) status.

		N	%
GSC	6	30	56.6%
	7	19	35.8%
	8	1	1.9%
	9	3	5.7%
Grade Group	1	30	56.6%
	2	9	16.9%
	3	10	18.9%
	4	1	1.9%
	5	3	5.7%
TNM	T2N0Mx	35	66.0%
	T3N0Mx	15	28.3%
	T3N1Mx	3	5.7%
T	2	35	66.0%
	3	18	34.0%
N	0	50	94.3%
	1	3	5.7%
Surgical margins	Negative	40	75.5%
	Positive	13	24.5%

**Table 2:** Periacinar retraction clefting groups’ distribution according to GSC in prostatic adenocarcinoma.

Prostatic Adenocarcinoma		GSC							
		6		7		8		9	
		No.	%	No.	%	No.	%	No.	%
Periacinar clefting	1	2	6.67%	3	15.79%	1	33.3%	3	100%
	2	2	6.67%	14	73.69%	0	0	0	0
	3	26	86.66%	2	10.52%	2	66.7%	0	0

**Table 3:** Periacinar retraction clefting in prostatic adenocarcinoma and in the adjacent nonneoplastic prostatic tissue and the immunohistochemical staining of E-cadherin and β-catenin in prostatic adenocarcinoma and in the adjacent nonneoplastic prostatic tissue.

		N	%
Periacinar retraction clefting in prostatic adenocarcinoma	1	9	16.9%
	2	16	30.18%
	3	28	52.92%
Periacinar retraction clefting in the adjacent nonneoplastic prostatic tissue	1	32	60.42%
	2	12	22.6%
	3	9	16.98%
E-cadherin immunostaining in prostatic adenocarcinoma	Negative	36	67.94%
	Weak	13	24.52%
	Positive	4	7.54%
E-cadherin immunostaining in the adjacent nonneoplastic prostatic tissue	Negative	0	0.0%
	Weak	5	9.43%
	Positive	48	90.57%
β-catenin immunostaining in prostatic adenocarcinoma	Negative	10	18.86%
	Weak	15	28.4%
	Positive	28	52.83%
β-catenin immunostaining in the adjacent nonneoplastic prostatic tissue	Negative	0	0.0%
	Weak	4	7.55%
	Positive	49	92.45%

**Table 4:** Correlation of E-cadherin and β-catenin immunohistochemical expression with Gleason score (GSC), Grade Group, preoperative serum prostate-specific antigen (sPSA), surgical margin status, and the Tumor (T) stage and Node (N) status in prostatic adenocarcinoma. P (probability value) and N (number). Spearman and Kendall tau_b coefficients.

		E-Cadherin Immunostaining in Prostatic Adenocarcinoma	β-Catenin Immunostaining in Prostatic Adenocarcinoma
GSC	Correlation Coefficient	−0.323	−0.750
	P	0.025	0.031
	N	53	53
Grade Group	Correlation Coefficient *	−0.630	−0.560
	P	0.038	0.019
	N	53	53
sPSA	Correlation Coefficient	0.06	0.11
	P	0.999	0.998
	N	53	53
Surgical margins	Correlation Coefficient *	−0.550	−0.390
	P	0.345	0.293
	N	53	53
T	Correlation Coefficient *	−0.720	−0.460
	P	0.111	0.143
	N	53	53
N	Correlation Coefficient *	−0.970	−0.680
	P	0.696	0.545
	N	53	53

* Kendall tau_b coefficient.

**Table 5:** Correlation of expression of E-cadherin and β-catenin with periacinar retraction clefting in prostatic adenocarcinoma and in the adjacent nonneoplastic prostatic tissue. P (probability value) and N (number). Spearman correlation coefficients.

		Periacinar Clefting
E-cadherin immunostaining in prostatic adenocarcinoma	Correlation Coefficient	0.350
	P	0.010
	N	53
E-cadherin immunostaining in the adjacent nonneoplastic prostatic tissue	Correlation Coefficient	−0.31
	P	0.542
	N	53
β-catenin immunostaining in prostatic adenocarcinoma	Correlation Coefficient	0.340
	P	0.012
	N	53
β-catenin immunostaining in the adjacent nonneoplastic prostatic tissue	Correlation Coefficient	−0.06
	P	0.664
	N	53

## Data Availability

All original materials and data are available upon request.
